# Poly[bis­(μ_2_-3-carb­oxy­benzoato)bis­(dipyrido[3,2-*a*;2′,3′-*c*]phenazine)bis­(μ_3_-isophthalato)tricopper(II)]

**DOI:** 10.1107/S1600536811008993

**Published:** 2011-03-15

**Authors:** Xiao-Fang Wang

**Affiliations:** a Pharmaceutic College, Liaoning University, 110036 Shenyang, People’s Republic of China

## Abstract

In the title compound, [Cu_3_(C_8_H_4_O_4_)_2_(C_8_H_5_O_4_)_2_(C_18_H_10_N_4_)_2_]_*n*_, one Cu^II^ atom, located on an inversion center, is hexa­coordinated and shows a distorted octa­hedral coordination geometry, while the other Cu^II^ atom is penta­coordinated and exhibits a distorted square-pyramidal geometry. The Cu^II^ atoms are bridged by isophthalate and 3-carb­oxy­benzoate ligands, forming a chain structure along the *b* axis. Furthermore, the chains are linked by O—H⋯O hydrogen bonds, forming a layer parallel to the *ab* plane.

## Related literature

For related structures, see: Han & Ma (2006 ▶); He & Han (2006 ▶); Han *et al.* (2009 ▶).
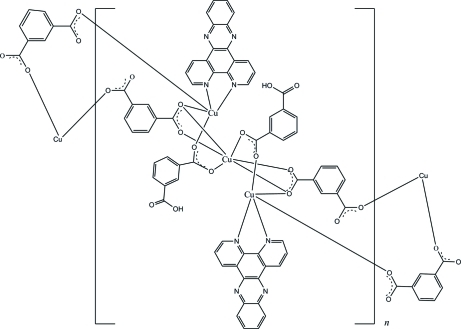


## Experimental

### 

#### Crystal data

[Cu_3_(C_8_H_4_O_4_)_2_(C_8_H_5_O_4_)_2_(C_18_H_10_N_4_)_2_]
*M* *_r_* = 1413.68
Triclinic, 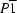

*a* = 10.6453 (12) Å
*b* = 11.6437 (13) Å
*c* = 12.3213 (14) Å
α = 103.186 (1)°
β = 93.712 (2)°
γ = 95.460 (2)°
*V* = 1474.3 (3) Å^3^
*Z* = 1
Mo *K*α radiation
μ = 1.16 mm^−1^
*T* = 293 K
0.37 × 0.33 × 0.27 mm

#### Data collection

Bruker APEX area-detector diffractometer
Absorption correction: multi-scan (*SADABS*; Sheldrick, 1996 ▶) *T*_min_ = 0.675, *T*_max_ = 0.747
7377 measured reflections
5099 independent reflections
4179 reflections with *I* > 2σ(*I*)
*R*_int_ = 0.018

#### Refinement

*R*[*F* ^2^ > 2σ(*F* ^2^)] = 0.035
*wR*(*F* ^2^) = 0.099
*S* = 1.05
5099 reflections
430 parameters
H-atom parameters constrained
Δρ_max_ = 0.32 e Å^−3^
Δρ_min_ = −0.31 e Å^−3^

### 

Data collection: *SMART* (Bruker, 2001 ▶); cell refinement: *SAINT* (Bruker, 2001 ▶); data reduction: *SAINT*; program(s) used to solve structure: *SHELXS97* (Sheldrick, 2008 ▶); program(s) used to refine structure: *SHELXL97* (Sheldrick, 2008 ▶); molecular graphics: *SHELXTL* (Sheldrick, 2008 ▶); software used to prepare material for publication: *SHELXTL*.

## Supplementary Material

Crystal structure: contains datablocks I, global. DOI: 10.1107/S1600536811008993/is2675sup1.cif

Structure factors: contains datablocks I. DOI: 10.1107/S1600536811008993/is2675Isup2.hkl

Additional supplementary materials:  crystallographic information; 3D view; checkCIF report

## Figures and Tables

**Table 1:** Hydrogen-bond geometry (Å, °)

*D*—H⋯*A*	*D*—H	H⋯*A*	*D*⋯*A*	*D*—H⋯*A*
O7—H7*B*⋯O4^i^	0.82	1.74	2.545 (3)	165

Symmetry code: (i) 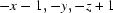.

**Fig. 1.**
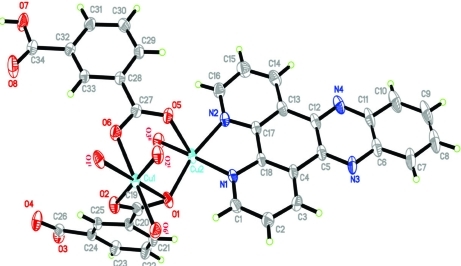
View of the structure of the title compound. Displacement ellipsoids are drawn at the 30% probability level. H atoms and the lattice water molecule have been omitted for clarity. [Symmetry codes: (i) -x, -y + 1, -z + 1; (ii) -x, -y, -z + 1].

**Fig. 2.**
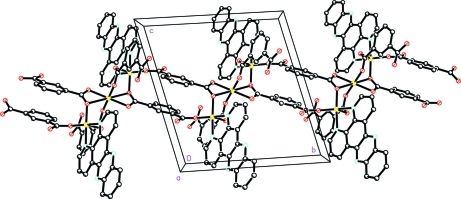
View of the one-dimensional double chain fragment of the title compound.

**Fig. 3.**
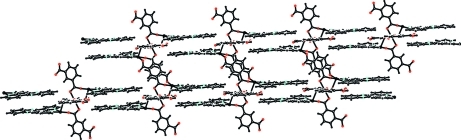
The layer structure.

**Fig. 4.**
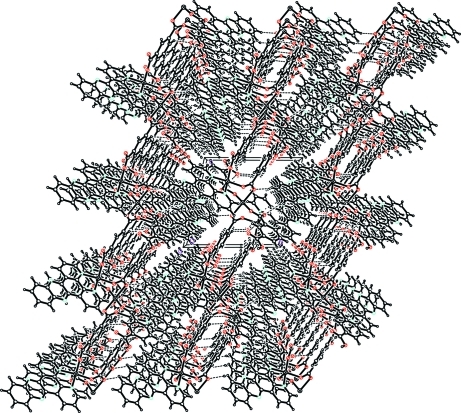
Packing diagram.

## supplementary crystallographic information

### Comment

Recently, several metal-organic complexes containing dipyridophenazine have been reported (Han & Ma, 2006; He & Han, 2006, Han *et al.*, 2009). We report here a new one-dimensional copper(II) coordination polymer constructed by Cu^II^ ions, dipyridophenazine (dppz) and isophthalic acid (H_2_ip), (I).

Complex (I) exhibits a one-dimensional double-chain structure in which the asymmetric unit consists of one and a half Cu^II^ ions, one ip^2-^, one Hip^-^ and one dppz ligand. Atom Cu1 is located on an inversion center and coordinated by six oxygen atoms, forming a slightly distorted octahedral geometry. On the other hand, atom Cu2 is coordinated by three oxygen atoms from two ip^2-^ ligands and one Hip^-^ ligand and two nitrogen atoms from a chelate dppz ligand to furnish a distorted square pyramidal geometry (Fig. 1). The carboxylate oxygen atoms *via* the *syn*-anti O,O'-bridges bridge three copper atoms (Cu1, Cu2 and Cu1^i^) to form a trinuclear [Cu_3_(ip)_2_(Hip)_2_(dppz)_2_] subunit, which are interconnected through the bridging ip^2-^ to form an infinite one-dimensional double chain (Fig. 2). These chains are further linked *via* strong hydrogen bonds between Hip^-^ and ip^-2^ ligands (Table 1), forming a layer structure.

### Experimental

A mixture of CuNO_3_.3H_2_O (0.5 mmol, 0.121 g), dipyridophenazine (0.5 mmol, 0.141 g), H_2_ip (0.5 mmol, 0.083 g) and water (10 ml) in a 23 ml Teflon reactor was heated at 453 K for six days and then cooled to room temperature at a rate of 5 K h^-1^ (yield 42%). Analysis for C_68_H_38_Cu_3_N_8_ (found/calc): C 58.18(57.77), H 2.86(2.71), N 5.83%(7.93%).

### Refinement

The H atoms of the aromatic rings were placed at calculated positions in the riding model approximation (C—H 0.93 Å) with their temperature factors were set to 1.2 times those of the equivalent isotropic temperature factors of the parent atoms. The hydroxy H atom was placed at calculated positions in the riding model approximation (O—H 0.82 Å) with their temperature factors were set to 1.5 times those of the equivalent isotropic temperature factors of the parent atoms.

### Crystal data

**Table:**

[Cu_3_(C_8_H_4_O_4_)_2_(C_8_H_5_O_4_)_2_(C_18_H_10_N_4_)_2_]	*Z* = 1
*M**_r_* = 1413.68	*F*(000) = 717
Triclinic, *P*1	*D*_x_ = 1.592 Mg m^−^^3^
Hall symbol: -P 1	Mo *K*α radiation, λ = 0.71073 Å
*a* = 10.6453 (12) Å	Cell parameters from 2406 reflections
*b* = 11.6437 (13) Å	θ = 2.4–20.1°
*c* = 12.3213 (14) Å	µ = 1.16 mm^−^^1^
α = 103.186 (1)°	*T* = 293 K
β = 93.712 (2)°	Block, green
γ = 95.460 (2)°	0.37 × 0.33 × 0.27 mm
*V* = 1474.3 (3) Å^3^	

### Data collection

**Table:**

Bruker APEX area-detector diffractometer	5099 independent reflections
Radiation source: fine-focus sealed tube	4179 reflections with *I* > 2σ(*I*)
graphite	*R*_int_ = 0.018
φ and ω scans	θ_max_ = 25.0°, θ_min_ = 1.8°
Absorption correction: multi-scan (*SADABS*; Sheldrick, 1996)	*h* = −9→12
*T*_min_ = 0.675, *T*_max_ = 0.747	*k* = −13→13
7377 measured reflections	*l* = −14→14

### Refinement

**Table:**

Refinement on *F*^2^	Primary atom site location: structure-invariant direct methods
Least-squares matrix: full	Secondary atom site location: difference Fourier map
*R*[*F*^2^ > 2σ(*F*^2^)] = 0.035	Hydrogen site location: inferred from neighbouring sites
*wR*(*F*^2^) = 0.099	H-atom parameters constrained
*S* = 1.05	*w* = 1/[σ^2^(*F*_o_^2^) + (0.0567*P*)^2^ + 0.1669*P*] where *P* = (*F*_o_^2^ + 2*F*_c_^2^)/3
5099 reflections	(Δ/σ)_max_ = 0.001
430 parameters	Δρ_max_ = 0.32 e Å^−^^3^
0 restraints	Δρ_min_ = −0.31 e Å^−^^3^

### Special details

**Table:**

Geometry. All e.s.d.'s (except the e.s.d. in the dihedral angle between two l.s. planes) are estimated using the full covariance matrix. The cell e.s.d.'s are taken into account individually in the estimation of e.s.d.'s in distances, angles and torsion angles; correlations between e.s.d.'s in cell parameters are only used when they are defined by crystal symmetry. An approximate (isotropic) treatment of cell e.s.d.'s is used for estimating e.s.d.'s involving l.s. planes.
Refinement. Refinement of *F*^2^ against ALL reflections. The weighted *R*-factor *wR* and goodness of fit *S* are based on *F*^2^, conventional *R*-factors *R* are based on *F*, with *F* set to zero for negative *F*^2^. The threshold expression of *F*^2^ > σ(*F*^2^) is used only for calculating *R*-factors(gt) *etc*. and is not relevant to the choice of reflections for refinement. *R*-factors based on *F*^2^ are statistically about twice as large as those based on *F*, and *R*- factors based on ALL data will be even larger.

### Fractional atomic coordinates and isotropic or equivalent isotropic displacement parameters (Å^2^)

**Table:**

	*x*	*y*	*z*	*U*_iso_*/*U*_eq_	
Cu1	0.0000	0.5000	0.5000	0.02989 (13)	
Cu2	0.16105 (3)	0.28114 (3)	0.30705 (3)	0.03371 (12)	
C1	0.3566 (3)	0.4952 (3)	0.3938 (2)	0.0432 (7)	
H1A	0.3151	0.5119	0.4588	0.052*	
C2	0.4679 (3)	0.5651 (3)	0.3878 (3)	0.0529 (8)	
H2A	0.4989	0.6283	0.4473	0.063*	
C3	0.5320 (3)	0.5404 (3)	0.2936 (3)	0.0499 (8)	
H3A	0.6081	0.5849	0.2895	0.060*	
C4	0.4814 (3)	0.4478 (2)	0.2042 (2)	0.0386 (7)	
C5	0.5409 (3)	0.4150 (3)	0.0987 (2)	0.0416 (7)	
C6	0.7044 (3)	0.4393 (3)	−0.0073 (3)	0.0559 (9)	
C7	0.8200 (3)	0.5009 (4)	−0.0229 (4)	0.0724 (12)	
H7A	0.8591	0.5637	0.0335	0.087*	
C8	0.8743 (4)	0.4683 (5)	−0.1208 (4)	0.0843 (15)	
H8A	0.9499	0.5099	−0.1311	0.101*	
C9	0.8181 (5)	0.3737 (5)	−0.2055 (4)	0.0913 (17)	
H9A	0.8576	0.3527	−0.2712	0.110*	
C10	0.7062 (4)	0.3103 (4)	−0.1950 (3)	0.0808 (13)	
H10A	0.6707	0.2461	−0.2518	0.097*	
C11	0.6448 (4)	0.3455 (4)	−0.0940 (3)	0.0617 (10)	
C12	0.4789 (3)	0.3224 (3)	0.0094 (2)	0.0444 (7)	
C13	0.3556 (3)	0.2621 (3)	0.0214 (2)	0.0417 (7)	
C14	0.2839 (3)	0.1781 (3)	−0.0637 (3)	0.0550 (9)	
H14A	0.3126	0.1582	−0.1345	0.066*	
C15	0.1709 (4)	0.1247 (3)	−0.0430 (3)	0.0631 (10)	
H15A	0.1212	0.0699	−0.1001	0.076*	
C16	0.1307 (3)	0.1528 (3)	0.0647 (3)	0.0484 (8)	
H16A	0.0555	0.1139	0.0791	0.058*	
C17	0.3064 (3)	0.2894 (2)	0.1253 (2)	0.0340 (6)	
C18	0.3685 (3)	0.3830 (2)	0.2154 (2)	0.0329 (6)	
C19	0.0936 (3)	0.3055 (2)	0.5382 (2)	0.0337 (6)	
C20	0.1180 (3)	0.1978 (2)	0.5788 (2)	0.0313 (6)	
C21	0.2345 (3)	0.1544 (3)	0.5679 (2)	0.0433 (7)	
H21A	0.2982	0.1943	0.5378	0.052*	
C22	0.2564 (3)	0.0524 (3)	0.6013 (3)	0.0540 (8)	
H22A	0.3356	0.0251	0.5965	0.065*	
C23	0.1596 (3)	−0.0095 (3)	0.6423 (3)	0.0443 (7)	
H23A	0.1735	−0.0796	0.6629	0.053*	
C24	0.0425 (3)	0.0324 (2)	0.6525 (2)	0.0315 (6)	
C25	0.0231 (2)	0.1374 (2)	0.6232 (2)	0.0302 (6)	
H25A	−0.0541	0.1679	0.6332	0.036*	
C26	−0.0649 (3)	−0.0392 (2)	0.6895 (2)	0.0357 (6)	
C27	−0.1168 (3)	0.3584 (2)	0.3066 (2)	0.0333 (6)	
C28	−0.2233 (2)	0.2739 (2)	0.2388 (2)	0.0310 (6)	
C29	−0.2136 (3)	0.2190 (3)	0.1282 (2)	0.0397 (7)	
H29A	−0.1427	0.2393	0.0933	0.048*	
C30	−0.3081 (3)	0.1344 (3)	0.0692 (2)	0.0473 (8)	
H30A	−0.3010	0.0972	−0.0051	0.057*	
C31	−0.4141 (3)	0.1051 (3)	0.1216 (2)	0.0462 (8)	
H31A	−0.4773	0.0471	0.0825	0.055*	
C32	−0.4265 (3)	0.1611 (2)	0.2310 (2)	0.0343 (6)	
C33	−0.3325 (2)	0.2459 (2)	0.2893 (2)	0.0322 (6)	
H33A	−0.3415	0.2848	0.3627	0.039*	
C34	−0.5433 (3)	0.1340 (3)	0.2864 (3)	0.0406 (7)	
N1	0.3072 (2)	0.40537 (19)	0.31041 (18)	0.0333 (5)	
N2	0.1971 (2)	0.23343 (19)	0.14657 (18)	0.0352 (5)	
N3	0.6503 (2)	0.4734 (2)	0.0899 (2)	0.0509 (7)	
N4	0.5305 (3)	0.2874 (2)	−0.0866 (2)	0.0565 (8)	
O1	0.1657 (2)	0.33737 (16)	0.46839 (15)	0.0423 (5)	
O2	0.00556 (19)	0.36275 (15)	0.57337 (16)	0.0402 (5)	
O3	−0.0436 (2)	−0.14264 (17)	0.69704 (17)	0.0462 (5)	
O4	−0.1638 (2)	0.00500 (19)	0.7081 (2)	0.0590 (6)	
O5	−0.01211 (18)	0.36972 (17)	0.26738 (16)	0.0397 (5)	
O6	−0.14098 (18)	0.41203 (17)	0.40347 (16)	0.0419 (5)	
O7	−0.6232 (2)	0.0496 (2)	0.22334 (19)	0.0604 (6)	
H7B	−0.6875	0.0414	0.2554	0.091*	
O8	−0.5632 (2)	0.1898 (2)	0.3764 (2)	0.0778 (8)	

### Atomic displacement parameters (Å^2^)

**Table:**

	*U*^11^	*U*^22^	*U*^33^	*U*^12^	*U*^13^	*U*^23^
Cu1	0.0317 (3)	0.0222 (2)	0.0369 (3)	−0.00111 (18)	0.01068 (19)	0.00878 (18)
Cu2	0.0394 (2)	0.02654 (18)	0.0388 (2)	0.00076 (14)	0.01725 (15)	0.01257 (14)
C1	0.0505 (19)	0.0445 (17)	0.0344 (15)	−0.0025 (14)	0.0111 (13)	0.0101 (13)
C2	0.054 (2)	0.0550 (19)	0.0457 (18)	−0.0133 (16)	0.0054 (15)	0.0108 (15)
C3	0.0380 (18)	0.056 (2)	0.057 (2)	−0.0101 (15)	0.0074 (15)	0.0230 (16)
C4	0.0343 (16)	0.0440 (16)	0.0462 (16)	0.0057 (13)	0.0133 (13)	0.0249 (14)
C5	0.0374 (17)	0.0481 (17)	0.0520 (18)	0.0130 (14)	0.0196 (14)	0.0301 (15)
C6	0.047 (2)	0.070 (2)	0.075 (2)	0.0265 (18)	0.0321 (18)	0.051 (2)
C7	0.048 (2)	0.095 (3)	0.107 (3)	0.029 (2)	0.041 (2)	0.073 (3)
C8	0.066 (3)	0.110 (4)	0.119 (4)	0.043 (3)	0.058 (3)	0.086 (3)
C9	0.094 (4)	0.120 (4)	0.108 (4)	0.070 (3)	0.081 (3)	0.082 (3)
C10	0.092 (3)	0.096 (3)	0.084 (3)	0.049 (3)	0.062 (3)	0.051 (2)
C11	0.065 (2)	0.078 (3)	0.071 (2)	0.043 (2)	0.048 (2)	0.052 (2)
C12	0.0519 (19)	0.0472 (18)	0.0474 (17)	0.0218 (15)	0.0256 (15)	0.0263 (14)
C13	0.0518 (19)	0.0378 (16)	0.0420 (16)	0.0149 (14)	0.0203 (14)	0.0147 (13)
C14	0.072 (2)	0.0530 (19)	0.0407 (17)	0.0107 (18)	0.0217 (16)	0.0071 (15)
C15	0.079 (3)	0.055 (2)	0.0470 (19)	−0.0061 (19)	0.0105 (18)	−0.0009 (16)
C16	0.051 (2)	0.0428 (17)	0.0494 (18)	−0.0013 (15)	0.0114 (15)	0.0078 (14)
C17	0.0363 (15)	0.0319 (14)	0.0389 (15)	0.0107 (12)	0.0121 (12)	0.0137 (12)
C18	0.0344 (15)	0.0345 (14)	0.0365 (14)	0.0088 (12)	0.0152 (12)	0.0172 (12)
C19	0.0435 (17)	0.0258 (13)	0.0321 (14)	−0.0035 (12)	0.0084 (12)	0.0091 (11)
C20	0.0376 (16)	0.0265 (13)	0.0312 (13)	−0.0011 (11)	0.0099 (11)	0.0095 (11)
C21	0.0376 (17)	0.0413 (16)	0.0570 (18)	0.0013 (13)	0.0190 (14)	0.0211 (14)
C22	0.0363 (18)	0.055 (2)	0.083 (2)	0.0139 (15)	0.0174 (16)	0.0352 (18)
C23	0.0444 (18)	0.0353 (15)	0.0613 (19)	0.0061 (14)	0.0102 (15)	0.0261 (14)
C24	0.0364 (15)	0.0278 (13)	0.0312 (13)	−0.0030 (12)	0.0057 (11)	0.0108 (11)
C25	0.0294 (14)	0.0276 (13)	0.0341 (14)	0.0014 (11)	0.0090 (11)	0.0075 (11)
C26	0.0409 (17)	0.0298 (14)	0.0359 (15)	−0.0063 (13)	0.0060 (12)	0.0105 (11)
C27	0.0352 (16)	0.0281 (13)	0.0416 (15)	0.0047 (12)	0.0060 (12)	0.0174 (12)
C28	0.0303 (14)	0.0282 (13)	0.0382 (14)	0.0069 (11)	0.0064 (11)	0.0126 (11)
C29	0.0363 (16)	0.0454 (16)	0.0415 (16)	0.0074 (13)	0.0128 (13)	0.0148 (13)
C30	0.0478 (19)	0.0555 (19)	0.0339 (15)	0.0053 (16)	0.0089 (13)	−0.0006 (14)
C31	0.0357 (17)	0.0527 (19)	0.0425 (17)	−0.0035 (14)	0.0029 (13)	−0.0012 (14)
C32	0.0297 (15)	0.0330 (14)	0.0418 (15)	0.0050 (12)	0.0054 (12)	0.0106 (12)
C33	0.0323 (15)	0.0304 (13)	0.0359 (14)	0.0059 (12)	0.0083 (12)	0.0093 (11)
C34	0.0343 (16)	0.0381 (16)	0.0503 (18)	0.0022 (13)	0.0097 (13)	0.0111 (14)
N1	0.0358 (13)	0.0353 (12)	0.0336 (12)	0.0039 (10)	0.0114 (10)	0.0156 (10)
N2	0.0382 (14)	0.0292 (12)	0.0411 (13)	0.0039 (10)	0.0139 (11)	0.0115 (10)
N3	0.0365 (14)	0.0626 (17)	0.0699 (18)	0.0120 (13)	0.0254 (13)	0.0408 (14)
N4	0.0676 (19)	0.0626 (18)	0.0550 (16)	0.0283 (15)	0.0374 (15)	0.0284 (14)
O1	0.0570 (13)	0.0348 (10)	0.0387 (11)	−0.0043 (9)	0.0203 (10)	0.0155 (9)
O2	0.0451 (12)	0.0292 (10)	0.0535 (12)	0.0070 (9)	0.0191 (10)	0.0194 (9)
O3	0.0503 (13)	0.0325 (11)	0.0615 (13)	−0.0037 (9)	0.0128 (10)	0.0240 (9)
O4	0.0437 (14)	0.0463 (13)	0.0969 (18)	0.0011 (11)	0.0299 (12)	0.0322 (12)
O5	0.0299 (11)	0.0405 (11)	0.0540 (12)	0.0025 (9)	0.0118 (9)	0.0201 (9)
O6	0.0375 (11)	0.0404 (11)	0.0441 (11)	−0.0052 (9)	0.0104 (9)	0.0045 (9)
O7	0.0341 (12)	0.0674 (15)	0.0715 (15)	−0.0148 (11)	0.0157 (11)	0.0052 (12)
O8	0.0653 (17)	0.0801 (18)	0.0713 (17)	−0.0248 (14)	0.0400 (14)	−0.0123 (14)

### Geometric parameters (Å, °)

**Table:**

Cu1—O6^i^	1.9070 (19)	C15—H15A	0.9300
Cu1—O6	1.9070 (19)	C16—N2	1.322 (4)
Cu1—O2	2.0112 (17)	C16—H16A	0.9300
Cu1—O2^i^	2.0112 (17)	C17—N2	1.349 (3)
Cu1—O1	2.690 (2)	C17—C18	1.440 (4)
Cu1—O1^i^	2.690 (2)	C18—N1	1.362 (3)
Cu2—O3^ii^	1.9334 (18)	C19—O2	1.245 (3)
Cu2—O1	1.9417 (18)	C19—O1	1.282 (3)
Cu2—N2	2.001 (2)	C19—C20	1.491 (4)
Cu2—N1	2.013 (2)	C20—C21	1.386 (4)
Cu2—O5	2.2750 (19)	C20—C25	1.388 (3)
C1—N1	1.327 (4)	C21—C22	1.378 (4)
C1—C2	1.389 (4)	C21—H21A	0.9300
C1—H1A	0.9300	C22—C23	1.388 (4)
C2—C3	1.372 (4)	C22—H22A	0.9300
C2—H2A	0.9300	C23—C24	1.385 (4)
C3—C4	1.392 (4)	C23—H23A	0.9300
C3—H3A	0.9300	C24—C25	1.381 (4)
C4—C18	1.388 (4)	C24—C26	1.507 (3)
C4—C5	1.471 (4)	C25—H25A	0.9300
C5—N3	1.316 (4)	C26—O4	1.227 (3)
C5—C12	1.428 (4)	C26—O3	1.269 (3)
C6—N3	1.356 (4)	C27—O5	1.250 (3)
C6—C11	1.409 (5)	C27—O6	1.270 (3)
C6—C7	1.412 (5)	C27—C28	1.497 (4)
C7—C8	1.362 (5)	C28—C29	1.382 (4)
C7—H7A	0.9300	C28—C33	1.400 (3)
C8—C9	1.390 (7)	C29—C30	1.379 (4)
C8—H8A	0.9300	C29—H29A	0.9300
C9—C10	1.370 (6)	C30—C31	1.388 (4)
C9—H9A	0.9300	C30—H30A	0.9300
C10—C11	1.437 (4)	C31—C32	1.377 (4)
C10—H10A	0.9300	C31—H31A	0.9300
C11—N4	1.354 (5)	C32—C33	1.374 (4)
C12—N4	1.331 (3)	C32—C34	1.499 (4)
C12—C13	1.461 (4)	C33—H33A	0.9300
C13—C14	1.388 (4)	C34—O8	1.193 (3)
C13—C17	1.396 (4)	C34—O7	1.304 (3)
C14—C15	1.367 (4)	O3—Cu2^ii^	1.9334 (18)
C14—H14A	0.9300	O7—H7B	0.8200
C15—C16	1.397 (4)		
			
O6^i^—Cu1—O6	180.0	C15—C16—H16A	119.2
O6^i^—Cu1—O2	91.80 (8)	N2—C17—C13	122.7 (3)
O6—Cu1—O2	88.20 (8)	N2—C17—C18	116.1 (2)
O6^i^—Cu1—O2^i^	88.20 (8)	C13—C17—C18	121.1 (3)
O6—Cu1—O2^i^	91.80 (8)	N1—C18—C4	122.7 (3)
O2—Cu1—O2^i^	180.000 (1)	N1—C18—C17	115.4 (2)
O6^i^—Cu1—O1	80.47 (7)	C4—C18—C17	121.8 (2)
O6—Cu1—O1	99.53 (7)	O2—C19—O1	121.8 (2)
O2—Cu1—O1	53.69 (6)	O2—C19—C20	119.8 (2)
O2^i^—Cu1—O1	126.31 (6)	O1—C19—C20	118.5 (2)
O6^i^—Cu1—O1^i^	99.53 (7)	C21—C20—C25	119.5 (2)
O6—Cu1—O1^i^	80.47 (7)	C21—C20—C19	119.9 (2)
O2—Cu1—O1^i^	126.31 (6)	C25—C20—C19	120.5 (2)
O2^i^—Cu1—O1^i^	53.69 (6)	C22—C21—C20	120.3 (3)
O1—Cu1—O1^i^	180.0	C22—C21—H21A	119.8
O3^ii^—Cu2—O1	93.55 (8)	C20—C21—H21A	119.8
O3^ii^—Cu2—N2	95.69 (9)	C21—C22—C23	119.7 (3)
O1—Cu2—N2	167.49 (9)	C21—C22—H22A	120.1
O3^ii^—Cu2—N1	169.76 (9)	C23—C22—H22A	120.1
O1—Cu2—N1	87.94 (8)	C24—C23—C22	120.4 (3)
N2—Cu2—N1	81.48 (9)	C24—C23—H23A	119.8
O3^ii^—Cu2—O5	86.18 (8)	C22—C23—H23A	119.8
O1—Cu2—O5	95.63 (8)	C25—C24—C23	119.4 (2)
N2—Cu2—O5	93.38 (8)	C25—C24—C26	120.3 (2)
N1—Cu2—O5	103.77 (8)	C23—C24—C26	120.2 (2)
N1—C1—C2	122.5 (3)	C24—C25—C20	120.5 (2)
N1—C1—H1A	118.8	C24—C25—H25A	119.8
C2—C1—H1A	118.8	C20—C25—H25A	119.8
C3—C2—C1	119.7 (3)	O4—C26—O3	126.0 (3)
C3—C2—H2A	120.2	O4—C26—C24	118.7 (2)
C1—C2—H2A	120.2	O3—C26—C24	115.3 (3)
C2—C3—C4	119.0 (3)	O5—C27—O6	124.7 (3)
C2—C3—H3A	120.5	O5—C27—C28	119.8 (2)
C4—C3—H3A	120.5	O6—C27—C28	115.4 (2)
C18—C4—C3	118.1 (3)	C29—C28—C33	119.2 (2)
C18—C4—C5	118.3 (3)	C29—C28—C27	121.1 (2)
C3—C4—C5	123.6 (3)	C33—C28—C27	119.6 (2)
N3—C5—C12	122.1 (3)	C30—C29—C28	120.6 (3)
N3—C5—C4	118.2 (3)	C30—C29—H29A	119.7
C12—C5—C4	119.7 (3)	C28—C29—H29A	119.7
N3—C6—C11	120.9 (3)	C29—C30—C31	119.4 (3)
N3—C6—C7	119.5 (4)	C29—C30—H30A	120.3
C11—C6—C7	119.6 (3)	C31—C30—H30A	120.3
C8—C7—C6	120.0 (4)	C32—C31—C30	120.7 (3)
C8—C7—H7A	120.0	C32—C31—H31A	119.6
C6—C7—H7A	120.0	C30—C31—H31A	119.6
C7—C8—C9	120.7 (4)	C33—C32—C31	119.8 (2)
C7—C8—H8A	119.6	C33—C32—C34	119.2 (2)
C9—C8—H8A	119.6	C31—C32—C34	121.0 (2)
C10—C9—C8	121.9 (4)	C32—C33—C28	120.3 (2)
C10—C9—H9A	119.0	C32—C33—H33A	119.9
C8—C9—H9A	119.0	C28—C33—H33A	119.9
C9—C10—C11	118.4 (5)	O8—C34—O7	124.1 (3)
C9—C10—H10A	120.8	O8—C34—C32	122.6 (3)
C11—C10—H10A	120.8	O7—C34—C32	113.1 (2)
N4—C11—C6	122.4 (3)	C1—N1—C18	118.0 (2)
N4—C11—C10	118.3 (4)	C1—N1—Cu2	129.29 (18)
C6—C11—C10	119.3 (4)	C18—N1—Cu2	112.39 (17)
N4—C12—C5	122.0 (3)	C16—N2—C17	119.0 (2)
N4—C12—C13	117.7 (3)	C16—N2—Cu2	128.0 (2)
C5—C12—C13	120.3 (2)	C17—N2—Cu2	113.01 (17)
C14—C13—C17	117.3 (3)	C5—N3—C6	116.9 (3)
C14—C13—C12	124.4 (3)	C12—N4—C11	115.7 (3)
C17—C13—C12	118.3 (3)	C19—O1—Cu2	130.45 (18)
C15—C14—C13	119.8 (3)	C19—O1—Cu1	75.87 (16)
C15—C14—H14A	120.1	Cu2—O1—Cu1	104.83 (8)
C13—C14—H14A	120.1	C19—O2—Cu1	108.67 (16)
C14—C15—C16	119.5 (3)	C26—O3—Cu2^ii^	129.9 (2)
C14—C15—H15A	120.3	C27—O5—Cu2	125.75 (16)
C16—C15—H15A	120.3	C27—O6—Cu1	116.10 (17)
N2—C16—C15	121.6 (3)	C34—O7—H7B	109.5
N2—C16—H16A	119.2		
			
N1—C1—C2—C3	−1.3 (5)	C31—C32—C34—O7	4.1 (4)
C1—C2—C3—C4	2.1 (5)	C2—C1—N1—C18	−0.8 (4)
C2—C3—C4—C18	−1.0 (4)	C2—C1—N1—Cu2	171.7 (2)
C2—C3—C4—C5	179.0 (3)	C4—C18—N1—C1	2.0 (4)
C18—C4—C5—N3	−177.4 (3)	C17—C18—N1—C1	−176.3 (2)
C3—C4—C5—N3	2.6 (4)	C4—C18—N1—Cu2	−171.7 (2)
C18—C4—C5—C12	3.1 (4)	C17—C18—N1—Cu2	10.0 (3)
C3—C4—C5—C12	−176.9 (3)	O3^ii^—Cu2—N1—C1	−109.2 (5)
N3—C6—C7—C8	−179.2 (3)	O1—Cu2—N1—C1	−10.6 (2)
C11—C6—C7—C8	−1.0 (5)	N2—Cu2—N1—C1	176.2 (3)
C6—C7—C8—C9	−0.9 (6)	O5—Cu2—N1—C1	84.7 (3)
C7—C8—C9—C10	0.7 (6)	O3^ii^—Cu2—N1—C18	63.6 (5)
C8—C9—C10—C11	1.4 (6)	O1—Cu2—N1—C18	162.24 (18)
N3—C6—C11—N4	1.9 (5)	N2—Cu2—N1—C18	−11.04 (18)
C7—C6—C11—N4	−176.2 (3)	O5—Cu2—N1—C18	−102.46 (18)
N3—C6—C11—C10	−178.8 (3)	C15—C16—N2—C17	−0.2 (5)
C7—C6—C11—C10	3.1 (5)	C15—C16—N2—Cu2	−176.8 (2)
C9—C10—C11—N4	176.1 (3)	C13—C17—N2—C16	−2.9 (4)
C9—C10—C11—C6	−3.2 (5)	C18—C17—N2—C16	175.0 (3)
N3—C5—C12—N4	2.3 (4)	C13—C17—N2—Cu2	174.2 (2)
C4—C5—C12—N4	−178.2 (3)	C18—C17—N2—Cu2	−7.8 (3)
N3—C5—C12—C13	−178.2 (3)	O3^ii^—Cu2—N2—C16	17.0 (3)
C4—C5—C12—C13	1.3 (4)	O1—Cu2—N2—C16	154.5 (3)
N4—C12—C13—C14	−5.8 (4)	N1—Cu2—N2—C16	−172.9 (3)
C5—C12—C13—C14	174.6 (3)	O5—Cu2—N2—C16	−69.5 (3)
N4—C12—C13—C17	173.6 (3)	O3^ii^—Cu2—N2—C17	−159.81 (18)
C5—C12—C13—C17	−6.0 (4)	O1—Cu2—N2—C17	−22.4 (5)
C17—C13—C14—C15	−1.0 (5)	N1—Cu2—N2—C17	10.27 (18)
C12—C13—C14—C15	178.4 (3)	O5—Cu2—N2—C17	113.69 (18)
C13—C14—C15—C16	−1.9 (5)	C12—C5—N3—C6	−2.0 (4)
C14—C15—C16—N2	2.5 (5)	C4—C5—N3—C6	178.5 (3)
C14—C13—C17—N2	3.5 (4)	C11—C6—N3—C5	0.0 (4)
C12—C13—C17—N2	−175.9 (2)	C7—C6—N3—C5	178.2 (3)
C14—C13—C17—C18	−174.3 (3)	C5—C12—N4—C11	−0.3 (4)
C12—C13—C17—C18	6.2 (4)	C13—C12—N4—C11	−179.9 (3)
C3—C4—C18—N1	−1.1 (4)	C6—C11—N4—C12	−1.7 (4)
C5—C4—C18—N1	178.9 (2)	C10—C11—N4—C12	179.0 (3)
C3—C4—C18—C17	177.0 (3)	O2—C19—O1—Cu2	−98.6 (3)
C5—C4—C18—C17	−3.0 (4)	C20—C19—O1—Cu2	82.2 (3)
N2—C17—C18—N1	−1.6 (3)	O2—C19—O1—Cu1	−1.0 (2)
C13—C17—C18—N1	176.4 (2)	C20—C19—O1—Cu1	179.8 (2)
N2—C17—C18—C4	−179.8 (2)	O3^ii^—Cu2—O1—C19	−13.4 (3)
C13—C17—C18—C4	−1.8 (4)	N2—Cu2—O1—C19	−151.0 (3)
O2—C19—C20—C21	−160.8 (3)	N1—Cu2—O1—C19	176.7 (3)
O1—C19—C20—C21	18.4 (4)	O5—Cu2—O1—C19	73.1 (2)
O2—C19—C20—C25	21.9 (4)	O3^ii^—Cu2—O1—Cu1	−97.32 (8)
O1—C19—C20—C25	−158.9 (2)	N2—Cu2—O1—Cu1	125.1 (4)
C25—C20—C21—C22	−0.3 (4)	N1—Cu2—O1—Cu1	92.82 (8)
C19—C20—C21—C22	−177.6 (3)	O5—Cu2—O1—Cu1	−10.81 (7)
C20—C21—C22—C23	2.4 (5)	O6^i^—Cu1—O1—C19	100.00 (16)
C21—C22—C23—C24	−1.8 (5)	O6—Cu1—O1—C19	−80.00 (16)
C22—C23—C24—C25	−0.9 (4)	O2—Cu1—O1—C19	0.63 (15)
C22—C23—C24—C26	175.8 (3)	O2^i^—Cu1—O1—C19	−179.37 (15)
C23—C24—C25—C20	3.0 (4)	O6^i^—Cu1—O1—Cu2	−131.28 (9)
C26—C24—C25—C20	−173.7 (2)	O6—Cu1—O1—Cu2	48.72 (9)
C21—C20—C25—C24	−2.4 (4)	O2—Cu1—O1—Cu2	129.35 (12)
C19—C20—C25—C24	174.9 (2)	O2^i^—Cu1—O1—Cu2	−50.65 (12)
C25—C24—C26—O4	−10.1 (4)	O1—C19—O2—Cu1	1.3 (3)
C23—C24—C26—O4	173.2 (3)	C20—C19—O2—Cu1	−179.46 (19)
C25—C24—C26—O3	169.0 (2)	O6^i^—Cu1—O2—C19	−77.44 (19)
C23—C24—C26—O3	−7.7 (4)	O6—Cu1—O2—C19	102.56 (19)
O5—C27—C28—C29	−8.2 (4)	O1—Cu1—O2—C19	−0.66 (16)
O6—C27—C28—C29	173.4 (2)	O1^i^—Cu1—O2—C19	179.34 (16)
O5—C27—C28—C33	169.0 (2)	O4—C26—O3—Cu2^ii^	21.2 (4)
O6—C27—C28—C33	−9.4 (3)	C24—C26—O3—Cu2^ii^	−157.74 (18)
C33—C28—C29—C30	−2.3 (4)	O6—C27—O5—Cu2	79.0 (3)
C27—C28—C29—C30	174.8 (3)	C28—C27—O5—Cu2	−99.2 (2)
C28—C29—C30—C31	0.4 (5)	O3^ii^—Cu2—O5—C27	39.4 (2)
C29—C30—C31—C32	1.1 (5)	O1—Cu2—O5—C27	−53.8 (2)
C30—C31—C32—C33	−0.7 (4)	N2—Cu2—O5—C27	134.8 (2)
C30—C31—C32—C34	177.1 (3)	N1—Cu2—O5—C27	−143.1 (2)
C31—C32—C33—C28	−1.2 (4)	O5—C27—O6—Cu1	−8.0 (3)
C34—C32—C33—C28	−179.1 (2)	C28—C27—O6—Cu1	170.25 (16)
C29—C28—C33—C32	2.7 (4)	O2—Cu1—O6—C27	−99.41 (19)
C27—C28—C33—C32	−174.5 (2)	O2^i^—Cu1—O6—C27	80.59 (19)
C33—C32—C34—O8	6.3 (5)	O1—Cu1—O6—C27	−46.71 (19)
C31—C32—C34—O8	−171.6 (3)	O1^i^—Cu1—O6—C27	133.29 (19)
C33—C32—C34—O7	−178.1 (3)		

Symmetry codes: (i) −*x*, −*y*+1, −*z*+1; (ii) −*x*, −*y*, −*z*+1.

### Hydrogen-bond geometry (Å, °)

**Table:**

*D*—H···*A*	*D*—H	H···*A*	*D*···*A*	*D*—H···*A*
O7—H7B···O4^iii^	0.82	1.74	2.545 (3)	165

Symmetry codes: (iii) −*x*−1, −*y*, −*z*+1.
